# Hot Spots and Their Contribution to the Self-Assembly of the Viral Capsid: In Silico Prediction and Analysis

**DOI:** 10.3390/ijms20235966

**Published:** 2019-11-27

**Authors:** Armando Díaz-Valle, José Marcos Falcón-González, Mauricio Carrillo-Tripp

**Affiliations:** 1 Biomolecular Diversity Laboratory, Centro de Investigación y de Estudios Avanzados del Instituto Politécnico Nacional Unidad Monterrey, Vía del Conocimiento 201, Parque PIIT, C.P. 66600 Apodaca, Nuevo León, Mexico; diaz.armando21@gmail.com; 2 Unidad Profesional Interdisciplinaria de Ingeniería Campus Guanajuato, Instituto Politécnico Nacional, Av. Mineral de Valenciana No. 200, Col. Fraccionamiento Industrial Puerto Interior, C.P. 36275 Silao de la Victoria, Guanajuato, Mexico; profymmarcos@gmail.com

**Keywords:** free energy, structural conservation, functional dimer, protein–protein interaction, site-directed mutagenesis, binding free energy, molecular dynamics, alanine-scanning

## Abstract

The viral capsid is a macromolecular complex formed by a defined number of self-assembled proteins, which, in many cases, are biopolymers with an identical amino acid sequence. Specific protein–protein interactions (PPI) drive the capsid self-assembly process, leading to several distinct protein interfaces. Following the PPI hot spot hypothesis, we present a conservation-based methodology to identify those interface residues hypothesized to be crucial elements on the self-assembly and thermodynamic stability of the capsid. We validate the predictions through a rigorous physical framework which integrates molecular dynamics simulations and free energy calculations by Umbrella sampling and the potential of mean force using an all-atom molecular representation of the capsid proteins of an icosahedral virus in an explicit solvent. Our results show that a single mutation in any of the structure-conserved hot spots significantly perturbs the quaternary protein–protein interaction, decreasing the absolute value of the binding free energy, without altering the protein’s secondary nor tertiary structure. Our conservation-based hot spot prediction methodology can lead to strategies to rationally modulate the capsid’s thermodynamic properties.

## 1. Introduction

The viral capsid is the archetypal molecular system of protein self-assembly and an excellent model for studying protein–protein interaction mechanisms that form symmetric closed shells [1]. The capsid is a macromolecular complex built by capsid proteins (CPs), which are biopolymers that in many cases have an identical amino acid sequence. Capsid formation occurs rapidly and spontaneously with a high degree of fidelity. The molecular mechanism followed by the CPs to form the capsid is not fully understood yet, due to the intrinsic complexity of the required protein–protein interfaces. There is evidence suggesting that the self-assembly process follows encoded signals in the sequence and structure of the CPs to guide the formation of the final virus particle. If correct, such molecular recognition signals are crucial elements for the initial nucleation and subsequent growth of the capsid. Therefore, accurate identification of those molecular signals, the so-called interface hot spots, will shed light on our understanding of the viral self-assembly process, or even macromolecular assembly in general.

### 1.1. Capsid Quaternary Structure

Viral capsids present either of two quaternary structures with basic symmetries: icosahedral (spherical or isotropic) or helical (rod-shaped, filamentous, or anisotropic). Icosahedral capsids are adopted by more than half of the virus families we currently know. In contrast, helical capsids are found in ~10% of virus families [2]. Watson and Crick proposed the basic principles for the construction of icosahedral viruses [3]. Then, Caspar and Klug developed the quasi-equivalence theory [4], which has been the foundation of modern structural virology.

In general, the residues of a protein in a monomeric state can be grouped according to their location in the native fold, namely, core residues or surface residues. In principle, the former are responsible for the tertiary structure, and the later are highly solvent-exposed. When a stable quaternary interaction takes place to form a protein complex, a third region is formed, i.e., the interface region. All residues in close contact between the interacting proteins are the interface residues of the complex.

**Figure 1:**
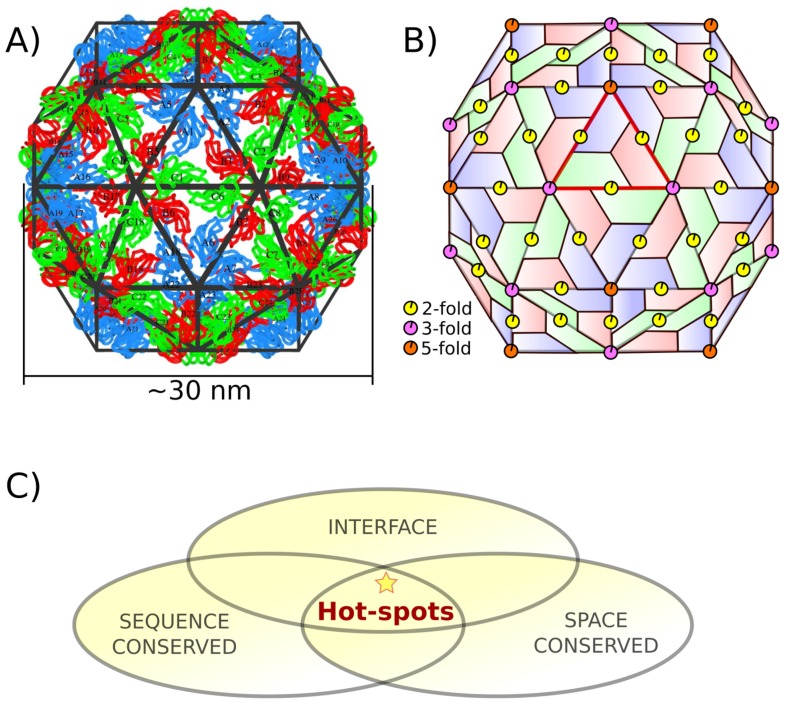
Quaternary structure of the capsid of an icosahedral virus. (**A**) An example of a T = 3 capsid (CCMV), which is formed by the assembly of 180 identical chemically identical proteins (CPs), or subunits, identified here by distinct labels ($A_{i},B_{i},$ and $C_{i}$). The corresponding color represents local arrangement, i.e., subunits of the same color have equivalent locations in the capsid. Each CP interacts and forms interfaces will all its surrounding neighbors. The conceptual icosahedral lattice is shown in black. (**B**) Each trapezoid represents a CP, or subunit, in the icosahedral arrangement. The location of the symmetry axes that define such geometry are shown. This architecture is characterized by having 60 equivalent triangular faces. One of them is highlighted (red), commonly referred to as the central icosahedral asymmetric unit. (**C**) Definition of the structure-conserved interface hot spots.

In particular, capsids are formed by a definite number of chemically identical proteins (CPs), also known as subunits. The CPs self-assemble spontaneously, yielding monodispersed particles (Figure 1A). In the case of spherical capsids, the arrangement of the CPs can be inscribed in an icosahedral lattice with symmetrical characteristics, as explained by the Caspar and Klug theory. In such a quaternary structure, each CP interacts and forms interfaces with all of its neighboring subunits. This geometrical arrangement creates a complex network of interface residues. To simplify the study of such network, its size can be reduced by taking advantage of the particle’s symmetries and focusing on just 1/60th of the capsid, commonly referred to as the icosahedral asymmetric unit (Figure 1B).

### 1.2. Protein–Protein Interface Hot Spot Prediction

Several works have proposed different strategies to predict the location of hot spots in a protein complex. Many of the available methods take advantage of the fact that, by definition, the contribution of each interface residue to the binding free energy is not homogeneous, i.e., some contribute significantly more than others. Commonly, an averaged energy-based alanine scanning mutagenesis approximation method is implemented for this purpose. Even though those methods can make fast calculations, one has to keep in mind that such approximations and physical assumptions will not necessarily produce a reliable result. Examples of this type of predictors are ROBBETA [5], FoldX [6], SpotOn [7], and iPPHOT [8]. Other approaches have opted for machine-learning-based methods [9,10,11,12], molecular-dynamics-based methods [13,14], or combining solvent accessibility and inter-residue potentials [15]. Despite these efforts, there is still not a clear way nor strict rule to locate hot spots on a protein complex.

On a previous work, we reported a methodology to map the 3D spatial location of the interface residues involved in all quaternary interactions in a capsid into a 2D representation, or CapsidMap [16,17]. The motivation to build a CapsidMap of a virus was to quantitatively evaluate the quaternary structure similarity between two capsids through a metric (S-score). When comparing capsids of different viruses, we noted the existence of a set of interface residues for the icosahedral Nodaviridae virus family who were conserved not only in sequence but also in quaternary structure among all related members. Furthermore, those structure-conserved interface residues were found to form non-random patterns around the capsid’s symmetry axes. Therefore, we propose that other virus families could also present residues with structural conservation characteristics. As a conclusion, in that work, we hypothesized that those residues should be interface hot spots and might have a crucial role in the self-assembly mechanisms due to their evolutionary persistence.

This work has two goals. First, we formally present a conservation-based methodology to locate hot spots. The following three steps define the general pipeline for a given virus family or genus. (i) For each member, identify all the interface residues between capsid subunits; this step is highly simplified by the use of the asymmetric unit and the automatized tools on the VIPERdb Science Gateway [18]. Then, for the whole set, (ii) identify residues conserved in sequence by multiple sequence alignment (MSA). Finally, (iii) identify residues conserved in quaternary structure by multiple CapsidMaps alignment. The intersection between the three sets of residues are the predicted conservation-based hot spots of that family or genus (Figure 1C). This is an alternative to other methods, i.e., no energy calculations nor physical approximations are involved in the predictions. Here, we applied such methodology to the Bromoviridae icosahedral virus family.

Second, we tested our hypothesis by calculating the binding free energy change produced to the wild type complex when mutating each one of the predicted hot spots. We implemented a rigorous all-atom physical framework with explicit solvent and controlled thermodynamic conditions. Steered Molecular Dynamics (SMD) and Umbrella Sampling simulations were carried out to calculate the value of the binding free energy through the Potential of Mean Force (PMF). Our results show that the structure-conserved interface residues hypothesized to be hot spots do have a significant contribution to the complex formation, as opposed to other nonconserved interface residues.

## 2. Results

### 2.1. Hot Spot Prediction

#### 2.1.1. Sequence Conservation of Interface Residues

**Figure A2:**
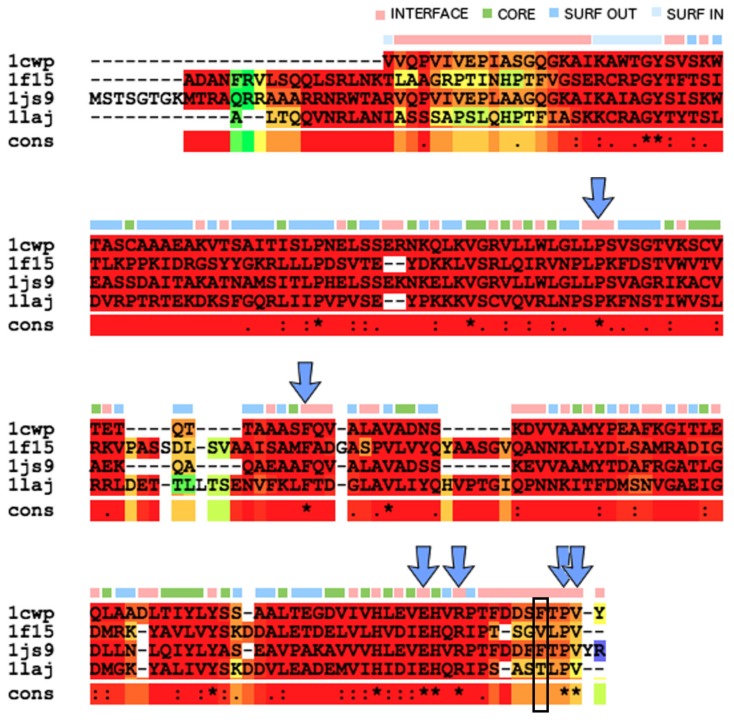
Multiple sequence alignment (MSA) of the Bromoviridae family members found in VIPERdb: Cowpea Chlorotic Mottle Virus (1cwp), Cucumber Mosaic Virus (1f15), Brome Mosaic Virus (1js9), and Tomato Aspermy Virus (1laj). The background color of the MSA represents tertiary structural similarity (low in green, medium in yellow, and high in red). Top row: location of each residue in the capsids quaternary structure, mapping the sequence into four distinct groups (interface: red; core: green: outer surface: blue; or inner surface: light blue-). Hot spots are indicated with arrows; P99, F120, E176, R179, P188, and V189. Position 186 is shown to be nonconserved interface.

**Figure A3:**
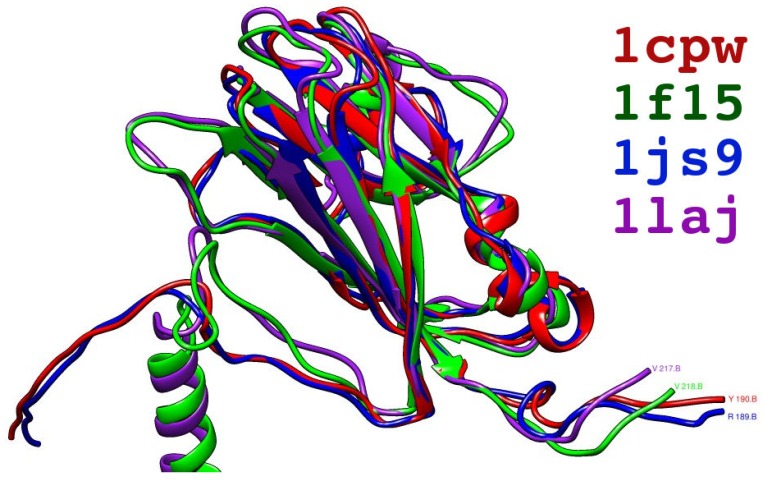
Multiple structure alignment of the CP of members of the Bromoviridae family: Cowpea Chlorotic Mottle Virus (1cwp in red), Cucumber Mosaic Virus (1f15 in green), Brome Mosaic Virus (1js9 in blue), and Tomato Aspermy Virus (1laj in purple).

We performed an MSA on the CPs of the Bromoviridae family members found in VIPERdb at the time of this writing, namely, Cowpea Chlorotic Mottle Virus (CCMV), Cucumber Mosaic Virus (CMV), Brome Mosaic Virus (BMV), and Tomato Aspermy Virus (TAV) (Appendix A, Figure A2). We corroborated the MSA by a tertiary structure alignment consensus made between the CP of the four viruses [19]. The CPs tertiary structure has a high degree of similarity between the four family members (Figure A3). However, sequence identity is low, suggesting a possible evolutionary structural convergence, as we had previously noted [20]. Sequence conservation is less than 10% of the total residues. Only half of the family sequence conserved residues are located in an interface: P99, F120, Y159, H172, E176, R179, P188, and V189 (CCMV sequence numbering).

#### 2.1.2. Space Conservation of Interface Residues

**Figure A4:**
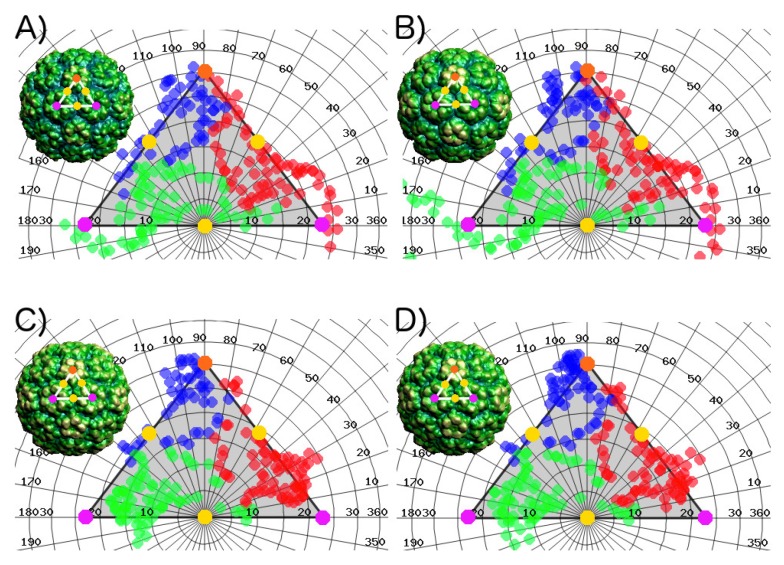
CapsidMaps of the interface residues of members of the Bromoviridae family, namely, (**A**) Cowpea Chlorotic Mottle Virus, (**B**) Brome Mosaic Virus, (**C**) Cucumber Mosaic Virus, and (**D**) Tomato Aspermy Virus. Each circle represents the location of an interface residue in ϕ−ψ space. The color used indicates the subunit to which the residue belongs (A in blue, B in red, and C in green). The icosahedral asymmetric unit used is indicated on the full capsid (insets), showing one 5-fold (orange), two 3-fold (magenta), and three 2-fold (yellow) symmetry axes. The 5-fold corresponds to ϕ=90−ψ=30, and the opposite 2-fold corresponds to ϕ=ψ=0.

The multiple quaternary structure alignment of the capsid of the four viruses was achieved through the CapsidMaps methodology [17]. A 2D depiction of the 3D position of the interface residues in the icosahedral asymmetric unit is built by projecting their space coordinates on a plane. Then, a conversion from Cartesian to Spherical coordinates is made. The ϕ-ψ angle space is used to generate a two-dimensional map of the quaternary patterns formed by all the interface residues in a particular capsid. The individual CapsidMaps of the four viruses studied in this work are shown in Figure A4. Conserved quaternary positions are readily identified when two or more CapsidMaps are compared. In the case of the Bromoviridae family, only six out of the eight interface sequence conserved residues are also conserved in quaternary structure.

#### 2.1.3. Hot Spot Predictions by the Structural Conservation Method

**Figure 2:**
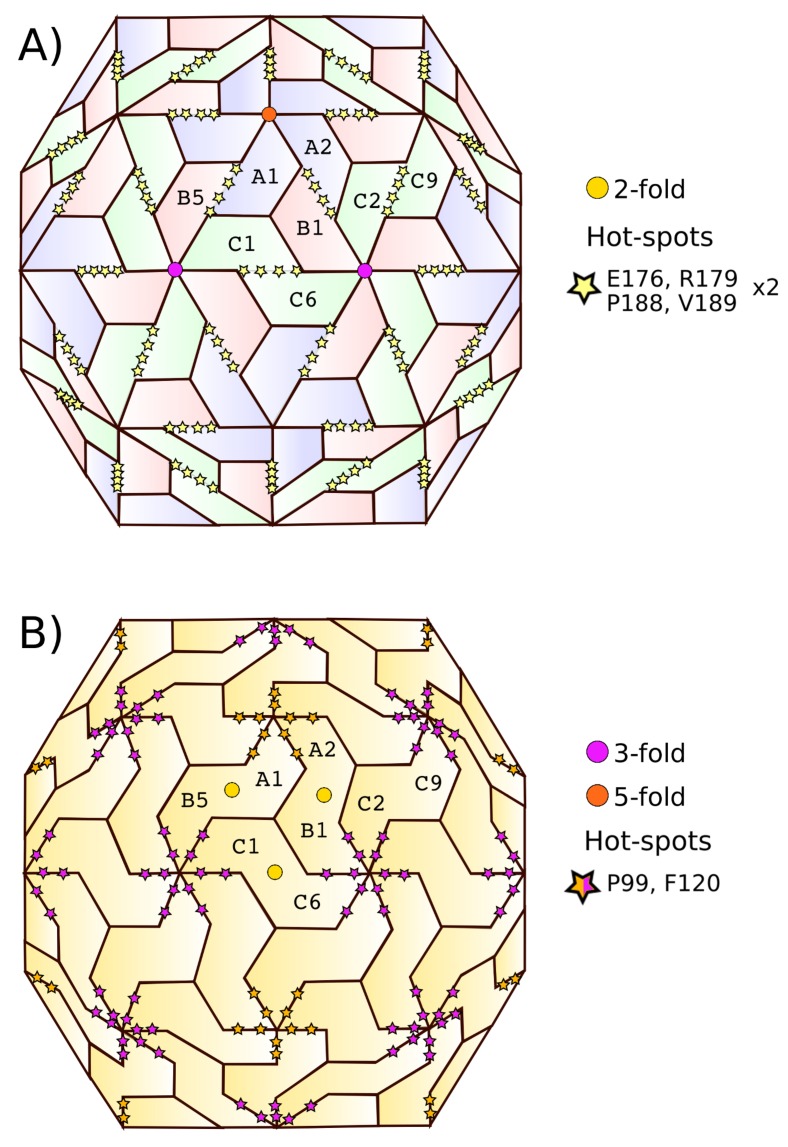
Mapping of the CCMV structure-conserved hot spots on the quaternary structure of the capsid. All hot spots were found to be closely related to a given symmetry axis: (**A**) 2-fold (E176, R179, P188, and V189) or (**B**) 3- and 5-fold (P99 and F120). Here, both representations of the capsid’s quaternary structure are equivalent, but (**B**) stresses the fact that the 2-fold-related dimers are the first oligomers formed on the kinetics of capsid self-assembly [21]. Symmetry axes are indicated for the central icosahedral asymmetric unit; only a few subunits are labeled.

**Figure A5:**
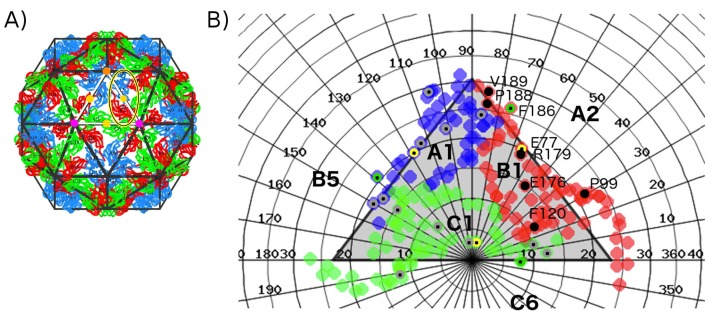
Multiple quaternary structure alignment of the Bromoviridae family members found in VIPERdb. The CCMV is used as the representative model. (**A**) Full capsid of the CCMV. The icosahedral asymmetric unit used in the analysis is indicated on white, showing one 5-fold (orange), two 3-fold (magenta), and three 2-fold (yellow) symmetry axes. The 5-fold corresponds to ϕ=90−ψ=30, and the opposite 2-fold corresponds to ϕ=ψ=0. All dimers related through a 2-fold axis are equivalent. One of them is highlighted, formed by the A2-B1 subunits. (**B**) CapsidMap of the CCMV interface residues, indicating the location of the hot spots for subunit B1 (black dots), A1 and C1 (gray–black dots) in ϕ−ψ space. Location of the interface residues in the control group is also indicated (yellow and green dots).

The conservation-based criteria identified a set of six residues, namely, P99, F120, E176, R179, P188, and V189 as hot spots (CCMV sequence numbering). Using the CCMV as a representative member, Figure A5 shows the spatial position of those six residues on a CapsidMap. To locate the specific CP–CP interfaces in the quaternary structure of the capsid where each hot spot is involved, we used VIPERdb’s contact tool (Virus Info Page-Annotations-Contact Tables-Which interfaces include a specific residue). Structure-conserved hot spots E176, R179, P188, and V189 were located in the interfaces made around a 2-fold axis, e.g., between subunits A1-B5, A2-B1, C1-C6, C2-C9, and so on. On the other hand, structure-conserved hot spots P99 and F120 were located in the interfaces made around a 3-fold or a 5-fold axis, e.g., between subunits A1-A2, B1-C2, B1-C6, B5-C1, and so on. The relationship between the subunit interfaces and the capsid’s symmetry folds, along with the location of the six structure-conserved hot spots of the Bromoviridae family is illustrated in Figure 2. Any dimer related by the same type of symmetry fold in the capsid quaternary structure is equivalent. Given that there is ample evidence that the 2-fold-related dimers, e.g., A2-B1, are the first step in the kinetics of capsid assembly in the case of CCMV [21] (i.e., dimers, pentamers of dimers, hexamers of dimers, etc.), we focused our efforts in the study of this particular type of CP–CP interface in this work.

#### 2.1.4. Hot Spot Predictions by Averaged Energy-Based Alanine Scanning Mutagenesis Approximation Methods

**Figure 3:**
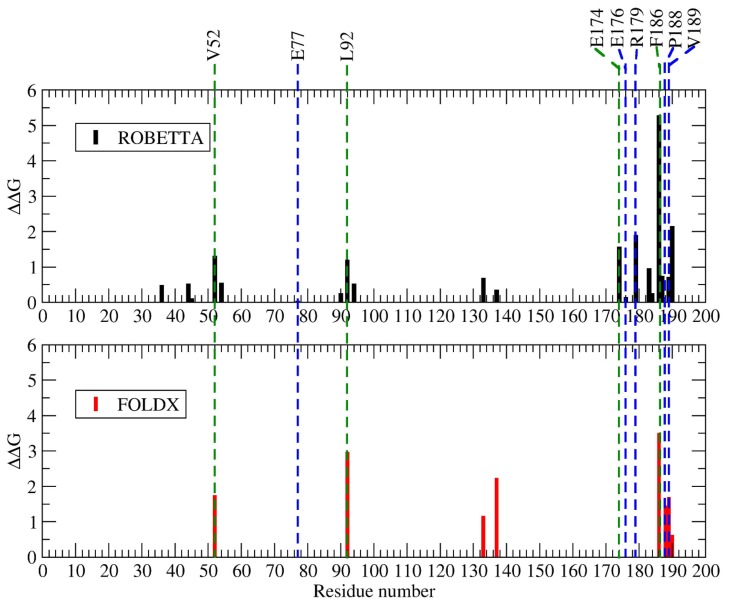
Computational energy-based alanine-scanning mutagenesis results from ROBBETA [5] (black), FoldX [6] (red), and hot spot predictions from SpotOn [7] (green) and this work (blue) of a 2-fold-related dimer interface. Interface residue E77 was randomly selected as an experimental control.

**Figure 4:**
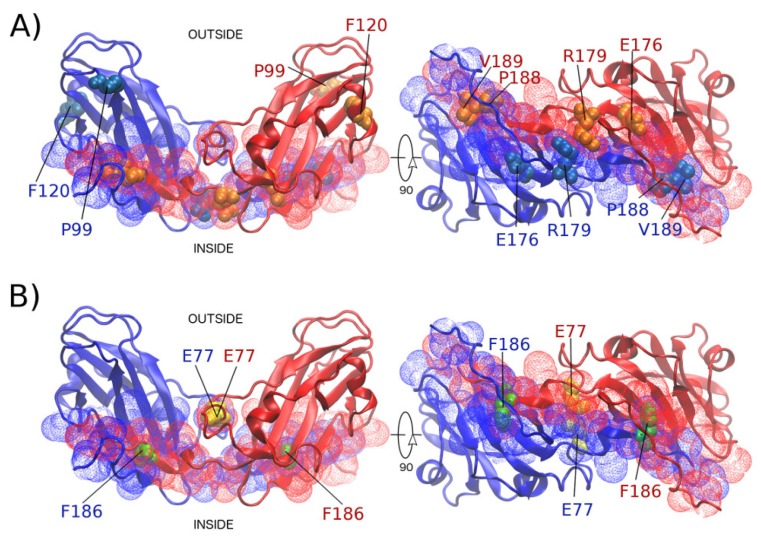
CCMV 2-fold-related dimer. (**A**) Location of the six structure-conserved hot spots in the quaternary structure (space-fill representation), showing subunit A2 in blue and subunit B1 in red (cartoon representation). The dimer interface residues are shown in mesh representation. A 90-degree rotation has been applied on the right to appreciate the interface better. (**B**) Location of the interface residues in the control group (using the same representations as before).

**Figure A6:**
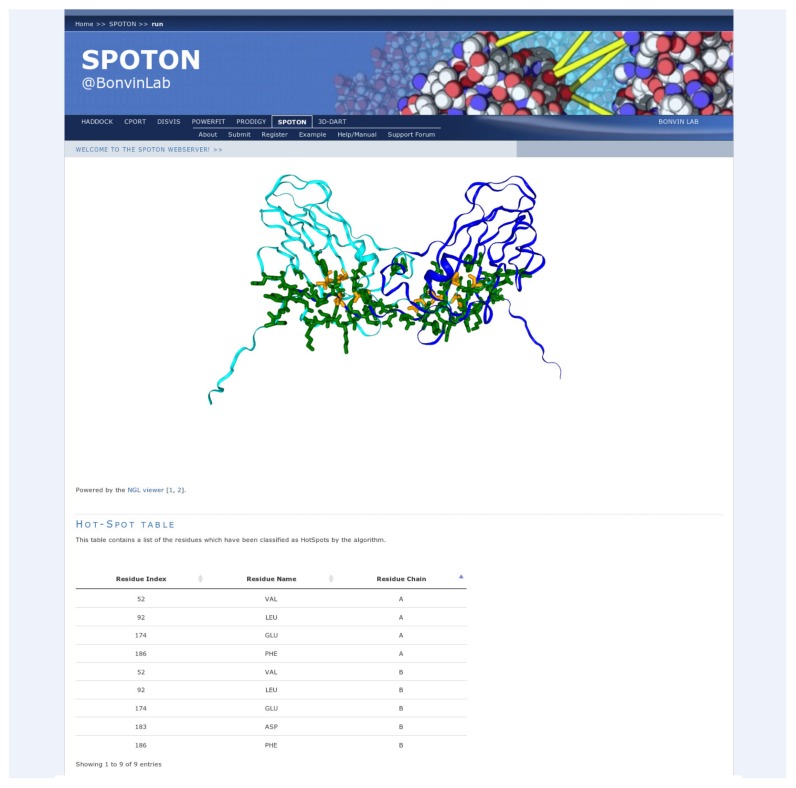
SpotOn [7] hot spot predictions for the structure of a representative 2-fold-related dimer.

**Figure A7:**
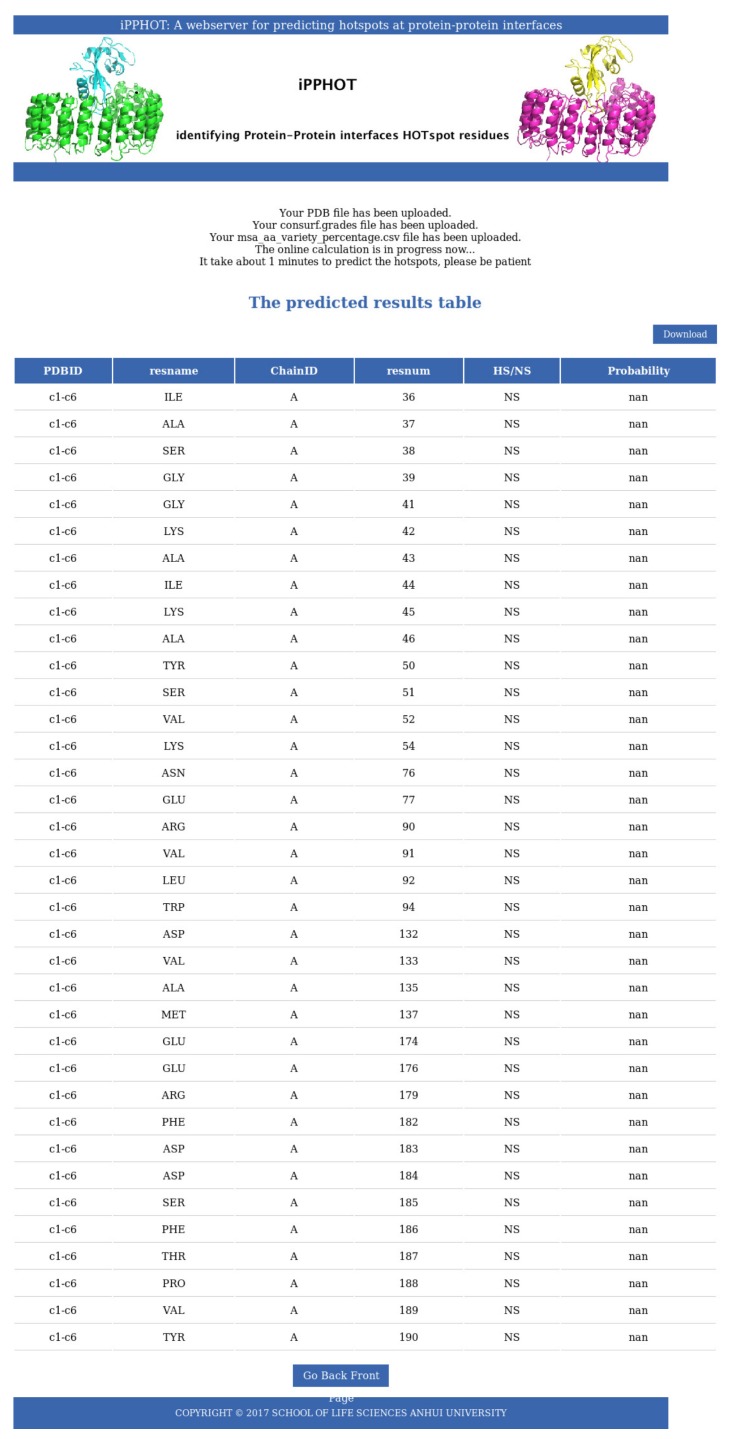
iPPHOT [8] hot spot predictions for the structure of a representative 2-fold-related dimer. All interface residues were predicted to be null-spots (NS).

To compare our methodology to other strategies, we used the averaged energy-based alanine scanning computational mutagenesis methods implemented in the ROBBETA, FoldX, SpotOn, and iPPHOT online tools to scan the interface of the 2-fold-related A2-B1 dimer of CCMV. The SpotOn tool only reports a list of potential hot spots (Figure A6). The iPPHOT tool was not able to find any hot spots on the A2-B1 protein complex (Figure A7). ROBBETTA and FoldX both report an approximation to the ΔΔG value in arbitrary units by interface residue. A comparison between all hot spot prediction results is presented in Figure 3. According to the averaged energy-based alanine scanning analyses, there is a consensus on the nonconserved interface residue F186 (Figure A2). In such an approximation, residue F186 shows a larger energy contribution than the rest of the interface residues, including the structure-conserved hot spots predicted with our methodology. Given this result, we included residue F186 in the following rigorous thermodynamic analysis. Furthermore, we randomly selected a nonconserved interface residue (E77) on the CCMV 2-fold-related dimer as an experimental control. The locations of all the residues analyzed in this work in the quaternary structure of the dimer are shown in Figure 4.

Note that a comparison of several physico-chemical characteristics shows that residue F186 stands out by having a low solvent accessible surface area (SASA) [22], large buried surface area (BSA) [22], lower association energy (AEne) [23] and solvation energy (SolvEne) [24], as well as a larger number of close intermolecular contacts (NumInt) with respect to the structure-conserved hot spots (Table 1). The fact that physico-chemical characteristics are the kind of averaged values used in the alanine-scanning strategies to approximately estimate ΔΔG is is probably the reason why all the averaged energy-based prediction tools pointed it out.

### 2.2. Hot Spot Validation Through a Rigorous Physical Framework

Alanine-scanning based strategies are limited by the implicit approximations and physical assumptions they are based on, which are used to make faster calculations. However, their predictions might not be accurate. The methodology we implemented to validate the hot spot predictions through the CP–CP binding free energy (ΔG is calculated with Umbrella sampling; PMF; and explicit solvent, temperature, and pressure control), although remarkably computationally more expensive, is a better approximation because it explicitly takes into consideration all interactions and thermodynamic effects present in the studied system. Therefore, the ΔG values obtained in such a rigorous theoretical framework are more reliable. The Molecular Dynamics trajectory data of all systems studied here can be accessed and visualized at the MDdb Science Gateway at http://www.md-db.org with Study ID 690002.

**Figure A8:**
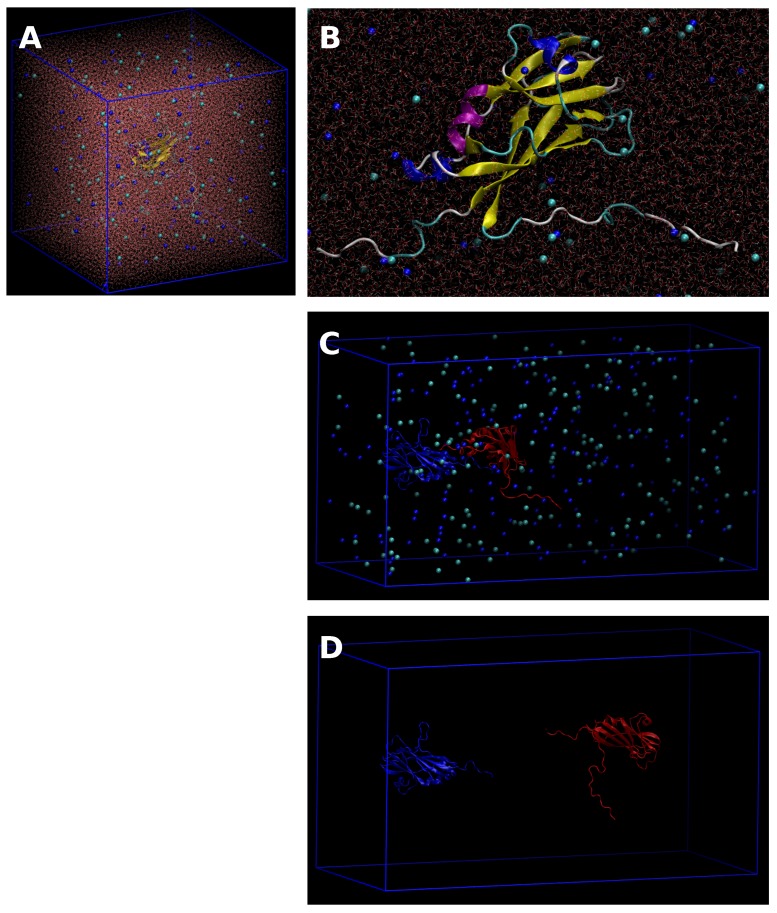
Simulation box of the Molecular Dynamics analysis of the CP of the CCMV in a monomer (**A**,**B**) or dimer (**C**,**D**) oligomerization state. (**A**) The CP (cartoon) was solvated with water (lines) and NaCl (spheres). (**B**) A closed-up view of the CP in the aqueous solution. Colors represent secondary structure and type of ion. (**C**) In the case of Steered Molecular Dynamics, the CP–CP dimer was also solvated with water (not shown) and NaCl is in a larger box. (**D**) COM–COM distance based configurations were used in Umbrella Sampling to build the PMF (water and NaCl not shown). Trajectory data available at MDdb.

**Figure A9:**
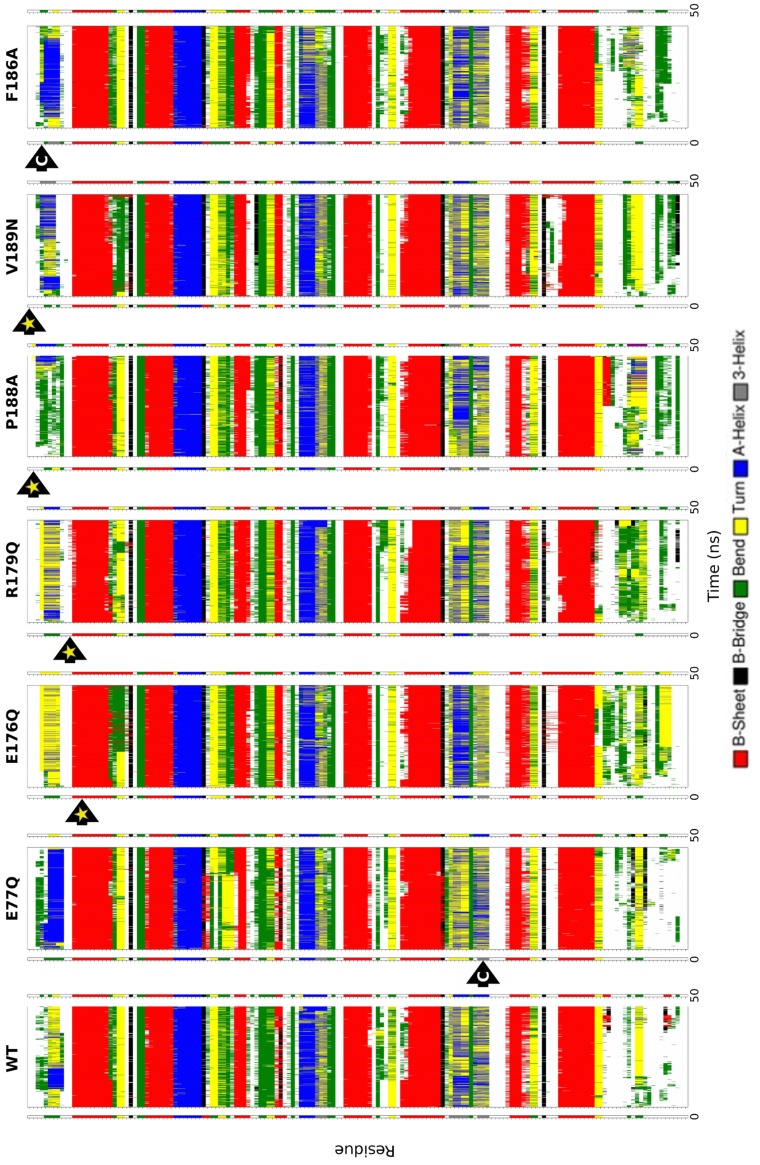
Analysis of monomer CP secondary structure relaxation by residue number over 50 ns of a Molecular Dynamics simulations for the seven CCMV CP variants studied in this work: wild type (WT), structure-conserved hot spots (stars), and control group (C). It can be seen that the point mutations (black arrows) do not disrupt the secondary structure of the CP. Trajectory data available at MDdb.

**Figure A10:**
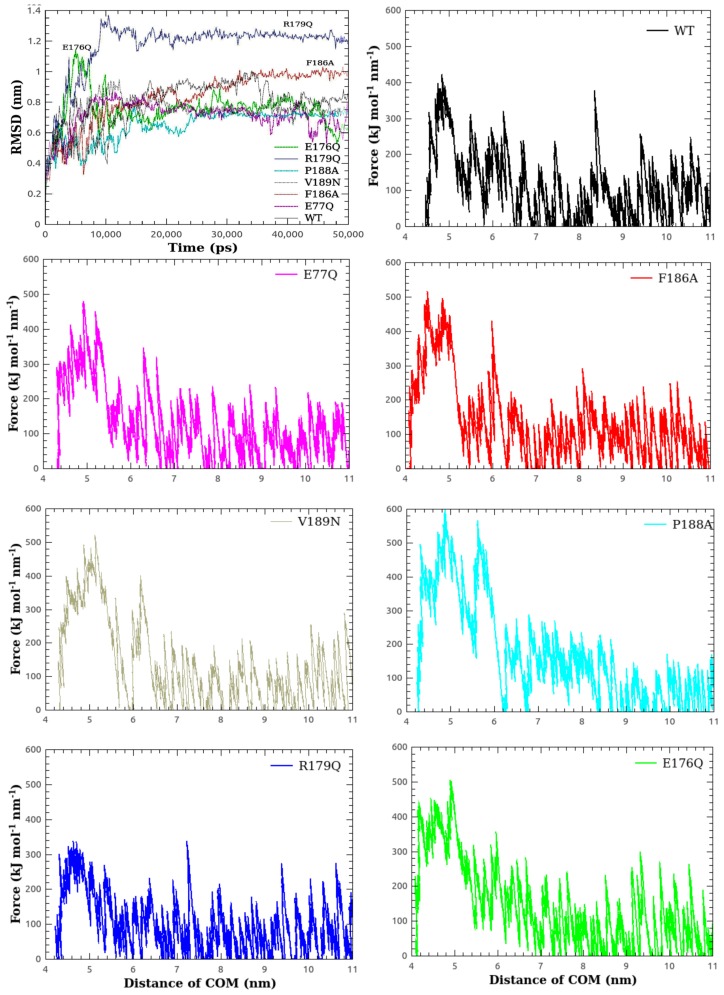
Analysis of monomer CP tertiary structure relaxation over 50 ns of a Molecular Dynamics simulations (RMSD vs. time) and 2-fold-related dimer breaking force (F vs. COM–COM distance) for the seven CCMV CP variants studied in this work. It can be seen that the point mutations do not disrupt the tertiary structure of the CP. Also, the force needed to break the protein complex remains approximately the same (≈400 kJ mol${}^{-1}$ nm${}^{-1}$). Trajectory data available at MDdb.

We mutated each structure-conserved hot spot and control set independently, producing seven variants. The rationale followed in the point mutations was to neutralize charges, change from nonpolar to polar, or from big to small side chain, to disrupt all possible wild type interactions. The final set was E176Q, R179Q, P188A, V189N, F186A, E77Q. A rigorous Molecular Dynamics analysis was performed on systems in thermodynamic equilibrium (NPT), where the proteins were completely solvated with explicit bulk water and NaCl at room temperature and pressure of 1 atm (Figure A8). The Umbrella methodology requires to sample conformations spaced along a reaction coordinate, which, in this case, was the distance between the center of mass (COM) of each subunit in the dimer. Therefore, SMD trajectories were generated for the wild type and the set of point mutations, starting from the homodimer complex and pulling the subunits away until their COMs were 10 nm apart. MD simulations showed that none of the point mutations disrupts the secondary nor the tertiary structure of the CCMV CP (Figure A9 and Figure A10, data available at MDdb). Furthermore, except for mutant R179Q, there is no change with respect to the wild type in the force needed to disassemble the protein complex (Figure A10).

**Figure 5:**
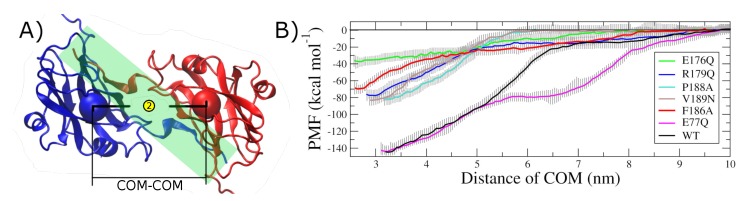
Potential of mean force (PMF) for the seven variants of the CCMV 2-fold-related dimer. (**A**) Quaternary structure of a 2-fold-related dimer (one subunit in blue and the other in red, cartoon representation). The reaction coordinate to generate the PMF profile was the distance between the center of mass (COM) of each subunit (spheres). The interface region is shaded. (**B**) PMF profiles as a function of subunit COM–COM distance for the wild type (WT), the point mutations in four structure-conserved hot spots (E176, R179, P188, and V189), and the two residues in the control group (E77 and F186).

The potential of mean force (PMF), as a function of subunit separation for the seven CCMV CP variants, was built from the Umbrella sampling and the Weighted Histogram Analysis Method (WHAM). The results are shown in Figure 5. The computational cost was 17,500 CPU hours, on average, for each one of the seven variants analyzed, totaling on an equivalent of 14 CPU years. As expected, the CP–CP distance in which the homodimer complex is thermodynamically stable corresponds to the global minimum in the PMF profile in all cases. Pulling the two subunits 10 nm away was enough to decrease their interaction energy to zero. This condition is sufficient to confidently assign the global minimum in the PMF profile as the binding free energy in each case.

Therefore, the change in the dimer interaction due to a point mutation relative to the wild type, ΔΔG, is the difference found between them. Table 2 shows the ΔΔG values for all the variants studied here. The randomly chosen nonconserved interface residue E77 (experimental control) produces a positive change of less than 1 kcal/mol when mutated. On the other hand, structure-conserved hot spot E176 produces a positive change of 108 kcal/mol. This value is close to 75% of the wild type binding free energy. The other three structure-conserved hot spots also produce a positive change, decreasing the thermodynamic stability of the 2-fold-related dimer by ~45%. Interface residue F186, although not a structure-conserved hot spot, produces a positive change close to 50%.

## 3. Discussion

In this work, we present a conservation-based strategy to identify protein–protein interface residues potentially relevant to the self-assembly of protein complexes, in particular, icosahedral viral capsids. Our findings provide evidence that a perturbation on any sequence-and-space conserved interface residue decreases the thermodynamic stability of the protein complex, in contrast to other nonconserved interface residues. Seemingly, such structure-conserved hot spots are important on the quaternary level, but not necessarily on the tertiary level. This statement might imply that a mutation on any of those conserved residues will not disrupt the protein fold but could prevent the CP–CP complex from forming.

On a previous study, we noted the existence of sequence-and-space conserved interface residues in the Nodaviridae family [16]. As in the case of the Bromoviridae family, the location of the conserved interface residues in the quaternary structure of the capsid was not randomly dispersed throughout the CP–CP interfaces, but forming patterns around the capsid’s symmetry axes. These findings are concomitant with the commonly accepted view of the capsid assembly kinetics. It has been shown that a nucleation seed needs to be formed to start the assembly process. At least in the case of the CCMV, this nucleation seed appears to be a pentamer of 2-fold-related dimers (POD) [25].

Hot spot identification is difficult because there is no apparent correlation between residue type or protein–protein interface composition with the way the complex is formed or the relative orientation between subunits (see Appendix A.1). Our hot spot prediction methodology is straightforward. Even though it is not based on an averaged energy calculation, we have shown, through the PMF, that the structure-conserved interface residues do have a substantial energy contribution to the stability of the protein complex in comparison to nonconserved residues. The fact that an interface residue has been conserved in sequence and space in the quaternary structure during evolution can be explained if that particular residue plays a crucial role in the molecular mechanism of self-assembly, either to direct the process or to provide a stabilization anchoring point between the interacting proteins. Therefore, structural conservation should be a better search criterion than, for example, averaged physico-chemical quantities or the number of intermolecular contacts (e.g., E176 vs. F186, Table 1).

We found that alanine-scanning- and structural conservation-based methodologies give different predictions in the case of CCMV of the Bromoviridae family. Residue F186 is not conserved, therefore it was not accounted for by the conservation-based method. On the other hand, none of the alanine-scanning predictors identified conserved residue E176 as a hot spot. The PMF of residue F186 shows a decrease of 50% in the binding free energy when mutated. However, the PMF of residue E176 shows a decrease of 75%. Through a fare comparison in this rigorous physical framework, E176 is clearly the most important hot spot residue, but it is missing from all other averaged energy-based prediction tools.

Currently, the averaged energy-based alanine-scanning strategy is the common way to search for hot spots. However, the energy function used is a rough approximation. This makes it a fast method to estimate an approximation to the energy contribution of all interface residues to the binding of a protein complex, one by one. However, such approximation will not necessarily provide an accurate description of the molecular interaction. For example, ROBETTA uses an effective energy function, i.e., making physical assumptions and averaging several contributions into one interaction term, to estimate an approximation to the energy change due to mutations to alanine on the isolated static molecular structure of the protein complex. In contrast, the rigorous thermodynamic analysis we implemented to validate the predictions (Umbrella sampling and PMF) employs a detailed description of all the bonded and non-bonded intra- and intermolecular interactions, explicitly taking into account the contribution of the solvent, temperature, and pressure over a length of time. The calculation of the binding free energy (PMF) was performed when the fully solvated homodimer was thermodynamically equilibrated. This is a closer, more reliable representation of in vitro conditions.

To compare both predictions, we used the method implemented in four available averaged energy-based hot spot prediction tools. Three of them identified nonconserved residue F186 as the one with a distinctive energy contribution to the complex. None of them picked out any of the six structure-conserved hot spots identified by the structure-based methodology. The fact that we found different predictions is not surprising since the fundamental hot spot search criteria used are not the same. Nonetheless, all methodologies (average-energy- or structure conservation-based) were in agreement concerning the interface residue E77 used as a positive control, whose PMF showed a contribution to the binding free energy close to zero (Figure 3).

The conservation-based prediction methodology has the limitation that a single structure is not enough to detect interface residues conserved at the quaternary level, i.e., the algorithm requires as many quaternary structures as possible. If a rigorous thermodynamic validation is required, the computation of the binding free energy (PMF) is costly and requires the use of high-performance computing. Nonetheless, that might not represent a problem nowadays with the increased access to supercomputing resources. All the computational packages used in our validation analysis are free and open source.

The hot spot prediction in silico thermodynamic validation by the binding free energy (PMF) shows that the structure-conserved interface residues do have a large contribution to the formation and stability of the protein complex. That result confirms our hypothesis; however, results of an in vitro biochemical analysis and biophysical validation are reported in a separate report. In view of our findings, a similar study of the thermodynamic contribution of structure-conserved hot spots P99 and F120 is in progress. Those residues were excluded from this work because they are involved in CP–CP interfaces different to the 2-fold-related. Most likely, their role will be in the formation of intermediate states in the process of capsid assembly, e.g., PODs.

## 4. Materials and Methods

### 4.1. Multiple Sequence Alignment

The Geneious R7 software was used to carry out a multiple sequence alignment (MSA) on a personal computer. The amino acid sequence of the CP of members of the Bromoviridae family whose molecular structure was available in the VIPERdb Science Gateway [18] were included.

### 4.2. Interface Residues and Quaternary Structure Alignment

We used the CapsidMaps [17] tool of VIPERdb to find the interface residues of members of the Bromoviridae family. The intrafamily structural alignments were carried out using the method previously described [16]. The cured crystallographic structures of the viruses were queried to produce a CapsidMap of the interface residues. The ϕ and ψ coordinates of each residue were recorded and then compared between viruses. An overlap threshold of 3${}^{\circ }$ in both angles was allowed to consider a conserved residue position in space.

### 4.3. Hot Spot In Silico Mutations

The 3D structure of the wild type (WT) 2-fold-related dimer was obtained from the Oligomer Generator tool of VIPERdb. Indications were followed, and the necessary parameters were introduced to obtain a file in PDB format with the atomic coordinates of the selected dimer (A2, B1). All hydrogen atoms were added with the WHAT IF Web Interface. The protonation state of histidine residues was set to physiological conditions (pH 7.0). Then, the Mutator Plugin tool of the Visual Molecular Dynamics software (VMD) [26] was used to generate one dimer with a point mutation for each one of the residues resulting from the structural conservation prediction. An additional point mutation was randomly selected from the A-B interface residues as an experimental control (E77).

### 4.4. Alanine Scanning Mutagenesis

A comparison with averaged energy-based hot spot prediction tools was made. We used the alanine scanning mutagenesis computational methods implemented in the ROBBETA [5], FoldX [6], SpotOn [7], and iPPHOT [8] (alignment created by ConSurf [27] using UNIREF90 and MAFFT) online tools. These are fast but coarse approaches for the prediction of energetically relevant amino acid residues in protein–protein interfaces. In all cases, the input was the 3D structure of the WT dimer in PDB format. The result was a list of residues predicted to significantly destabilize the interface when mutated to alanine, based on an approximated energy function.

### 4.5. Steered Molecular Dynamics

To accurately calculate the dimers binding free energy with a rigorous physical framework, a previously reported computational method using molecular dynamics was implemented [28]. We followed the same procedure for all the variants produced in the previous section, both in the monomer or dimer oligomerization state. The CHARMM27 all-atom force field (FF) plus CMAP for proteins was used to describe molecular interactions [29]. This FF was chosen because it was shown that substantial deviations from experimental backbone root-mean-square fluctuations and N–H NMR order parameters obtained in the MD trajectories are eliminated by the CMAP correction, therefore improving dynamical and structural properties of proteins. The dimers were solvated with liquid water using the TIP3P potential function [30].

All simulations were performed using the GROMACS 4.5.5 suite [31]. The monomers were placed in a cubic box, whereas the dimers were placed inside a rectangular box (Figure A8). In either case, the dimensions of the simulation box were chosen such that the minimum distance between any atom of the protein and the walls were no less than 1.0 nm, as well as to provide sufficient space for the pulling to take place along the x-axis. The empty volume was filled with water molecules. Also, Na${}^{+}$ and Cl${}^{-}$ ions were added in a proportion that would neutralize the overall charge of the system and obtain a final salt concentration of 100 mM. Bad contacts between any two atoms were removed by energy minimization of the whole system using the steepest descent algorithm with a force tolerance of 100.0 kJ/mol/nm and a 0.01 step size. Isochoric–isothermal (NVT) equilibration of solvent molecules to 300 K was performed for 100 ps, with the proteins heavy atoms being restrained by a harmonic potential with a force constant of 1000.0 kJ/mol/nm. A subsequent isobaric–isothermal (NPT) equilibration to adjust the system density was performed for another 100 ps at 1 bar.

Further structural equilibration simulations were carried out in the NPT ensemble at a temperature of 300 K and a pressure of 1 bar for 1 ns removing all position restraints. The temperature was maintained using the V-rescale thermostat with a coupling time constant of 0.1 ps. The dimer and solvent molecules were coupled to separate thermostats to avoid the hot solvent–cold solute issue. The pressure was regulated using the isotropic Parrinello–Rahman barostat with a coupling time of 2.0 ps and compressibility of 4.5 × 10${}^{-5}$ bar${}^{-1}$. Bonds involving hydrogen were constrained to their equilibrium values using the LINCS algorithm. The non-bonded interactions (Lennard–Jones and electrostatic) were truncated at 1.0 nm. Long-range electrostatic interactions beyond the cut-off distance were calculated using the particle mesh Ewald (PME) method, with a Fourier spacing of 0.16 nm and a cubic interpolation of order 4. A long-range analytic dispersion correction was also applied to both energy and pressure to account for the truncation of the Lennard–Jones interaction. The time-dependent dynamics of the system was evolved using the leap-frog integrator with a time step of 2 fs.

After full equilibration, position restraints were set again for subunit A only; therefore, using it as an immobile reference for the pulling simulations. For each of the dimer variants, subunit B was pulled away from subunit A along the x-axis over 2000 ps, using a spring constant of 2000 kJ mol${}^{-1}$ nm${}^{-2}$ and a pull rate of 0.0035 nm ps${}^{-1}$ (0.035 Å ps${}^{-1}$). A final center-of-mass (COM) distance of approximately 7 nm between subunits A and B was achieved.

### 4.6. Umbrella Sampling

From the previous SMD trajectories, snapshots were taken to generate the starting configurations for the Umbrella Sampling windows [32]. An asymmetric distribution of windows was used, such that the spacing was between 1.5 and 2 nm COM separation. Such spacing resulted in 42 windows per dimer. For each window, 5 ns of MD was performed for a total simulation time of 210 ns × 7 dimers utilized for Umbrella Sampling. Analysis of results was performed with the Weighted Histogram Analysis Method (WHAM) [33] for the generation of the Potential of Mean Force (PMF) profile as a function of the reaction coordinate (COM separation).

## Figures and Tables

**Table 1:** Approximation values of physico-chemical characteristics of the CCMV capsid hot spots. Solvent Accessible Surface Area (Å${}^{2}$), Association Energy (kcal/mol), Solvation Energy (kcal/mol), Buried Surface Area (Å${}^{2}$), and number of intermolecular distance-based close contacts.

Residue	SASA	AEne	SolvEne	BSA	NumInt
Proline P99 ^*a*^	9.70	−3.29	−2.03	141.20	5
Phenylalanine F120 ^*a*^	11.10	−0.73	−0.35	34.73	1
Glutamic Acid E176 ^*b*^	0.00	−0.16	−0.10	6.74	1
Arginine R179 ^*b*^	64.50	−2.80	0.32	138.64	3
Proline P188 ^*b*^	39.20	−5.03	−3.47	203.97	1
Valine V189 ^*b*^	28.70	−4.48	−2.92	187.98	4
Glutamic Acid E77 ^*c*^	65.50	−2.72	−0.54	112.78	1
Phenylalanine F186 ^*c*^	1.70	−9.58	−6.70	385.04	7

^*a*^ 3- and 5-fold interfaces. ^*b*^ 2-fold interface. ^*c*^ Control group, also in the 2-fold interface.

**Table 2:** Free energy of dimerization, ΔG, and change in the dimer interaction due to a point mutation, ΔΔG, relative to the wild type (WT) variant. All units in (kcal/mol).

Variant	Property	Mutant	Property	ΔG	ΔΔG
WT				−144.9 ± 4.9	0.0
Glutamic Acid E176 ^*a*^	Negative Charge	Glutamine Q	Neutral	−37.4 ± 5.3	107.6
Arginine R179 ^*a*^	Positive Charge	Glutamine Q	Neutral	−77.5 ± 6.1	67.5
Proline P188 ^*a*^	Special case	Alanine A	Small	−82.0 ± 5.8	63.0
Valine V189 ^*a*^	Nonpolar	Asparagine N	Polar	−83.2 ± 5.6	61.8
Glutamic Acid E77 ^*b*^	Negative Charge	Glutamine Q	Neutral	−144.1 ± 5.8	0.9
Phenylalanine F186 ^*b*^	Big	Alanine A	Small	−69.6 ± 5.9	75.4

^*a*^ 2-fold interactions. ^*b*^ Control group.

**Figure A1:**
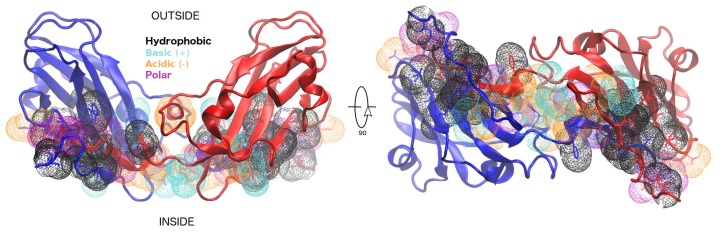
CCMV 2-fold-related homodimer. Front view of the complex of subunit A2 (blue) and subunit B1 (red) in cartoon representation. The outside of the capsid is on top and the interior in the bottom. The dimer interface residues are shown in mesh representation, color-coded to represent the individual physicochemical property (hydrophobic, polar, basic, or acid). A 90-degree rotation has been applied on the right to appreciate the interface better.

## Abbreviations

The following abbreviations are used in this manuscript.

CCMV	Cowpea Chlorotic Mottle Virus
CP	capsid protein
WT	wild type
COM	center-of-mass
MD	Molecular Dynamics
SMD	Steered Molecular Dynamics

## Appendix A. Supplementary Data

### Appendix A.1. Composition of the 2-Fold-Related Interface

The Cowpea Chlorotic Mottle Virus (CCMV) belongs to the Bromoviridae family. We use it as a representative member from this point on. The CCMV capsid protein residue composition is not homogeneous. An analysis of the CPs quaternary structure shows this holds for the interfaces too (Figure A1). The structural regions found in a protein, namely, interface, core, and surface, grouped by their physicochemical nature are summarized in Table A1 for the case of the CCMV CP. A proportion of 40–40–20 percentage is found for residues on the interface, surface, and core, respectively. Half the total residues are nonpolar, distributed in 20–20–10 proportion in the same order. Even though there is a couple of cysteines in the core, they are too far apart to form a disulfide bond. The small number of aromatic residues seem to be evenly distributed throughout the protein structure. However, a significantly larger number of charged residues are found in the interface with respect to any other structural region in this particular case.

Other physical properties have been used to characterize a protein. Some of these are the hydrophobicity (H) [34], the solvent accessible surface area (SASA) [22], the association energy (AEne) [23], the solvation energy (SolvEne) [24], and the buried surface area (BSA) [22]. We show a summary of these properties for the structural regions of the CCMV CP in Table A2, as reported on VIPERdb’s contact tables [18] (entry ID 1cwp). The total hydrophobicity by structural groups was estimated as the sum of the hydrophobicity index value of all residues in each group. As expected, the core and interface regions present large and equal values of hydrophobicity. Nonetheless, the surface exposed to the solvent also presents a large hydrophobicity. On the other hand, the surface in contact with the nucleic acids is rather hydrophilic. In terms of surface areas, the core region presents low values in both BSA and SASA. The outside surface presents low BSA but high SASA. The interface region has large values both in BSA and SASA. This observation could be related to the local flexibility of each region [35]. It is not surprising that most of the AEne come from the interface region.

**Table A1:** CCMV capsid protein residue composition by structural groups. Number of residues is displayed in percentage (%).

Group	Nonpolar	Polar	Negative	Positive	Total	CYS	TRP
Interface	33 (20)	14 (9)	12 (8)	10 (6)	69 (42)		W94
Dimer ^*a*^	14 (9)	5 (3)	5 (3)	3 (1)	27 (16)		W94
Core	22 (13)	8 (5)	2 (1)	1 (1)	33 (20)	C59, C108	
Surf-Out	31 (19)	20 (12)	2 (1)	2 (1)	55 (34)		W55
Surf-In	4 (2)	2 (1)	0 (0)	1 (1)	7 (4)		W47
Total	90 (54)	44 (27)	16 (10)	14 (9)	164 (100)		

^*a*^ Counted in Interface residues.

**Table A2:** CCMV capsid protein physical-chemical properties by structural groups. Hydrophobicity (Mehler scale), Solvent Accesible Surface Area (Å${}^{2}$), Association Energy (kcal/mol), Solvation Energy (kcal/mol), and Buried Surface Area (Å${}^{2}$).

Group	H	SASA	AEne	SolvEne	BSA
Interface	18.5	2382.3	−147.1	−51.4	6351.9
Dimer ^*a*^	8.6	864.2	−87.1	−34.4	3707.0
Core	19.2	78.4	−0.2	−0.1	8.9
Surf-Out	14.6	2853.5	−3.4	−0.8	171.1
Surf-In	2.2	604.3	−2.5	−1.2	118.4

^*a*^ Part of the Interface residues.
